# P-710. Epidemiology of RSV infections in a birth cohort of healthy children, PREVAIL Cohort 2017-2020

**DOI:** 10.1093/ofid/ofae631.906

**Published:** 2025-01-29

**Authors:** Maria Deza Leon, Shannon C Conrey, Monica Epperson, Melissa Coughlin, Allison R Cline, Claire Mattison, Daniel C Payne, Meredith L McMorrow, Ardythe L Morrow, Natalie J Thornburg, Mary A Staat

**Affiliations:** Children's Mercy Hospital, Overland Park, Kansas; University of Cincinnati College of Medicine, Cincinnati, Ohio; Centers for Disease Control and Prevention, Atlanta, Georgia; CDC, Atlanta, Georgia; University of Cincinnati College of Medicine, Cincinnati, Ohio; Centers for Disease Control, Atlanta, Georgia; CDC, Atlanta, Georgia; CDC/NCIRD/CORVD/SPB, Atlanta, GA; University of Cincinnati College of Medicine, Cincinnati, Ohio; Centers for Disease Control and Prevention, Atlanta, Georgia; Cincinnati Children’s Hospital Medical Center, Cincinnati, Ohio

## Abstract

**Background:**

Acute bronchiolitis due to respiratory syncytial virus (RSV) is the leading cause of hospitalizations in infants in the United States. Most available data on RSV epidemiology were obtained from hospital cohorts or medically attended illnesses, focusing on symptomatic children. We examined the PREVAIL Cohort (2017–2020) of healthy, term infants in Cincinnati, Ohio to understand the epidemiology of RSV infections in a birth cohort under weekly surveillance.

Table 1

**Figure:**
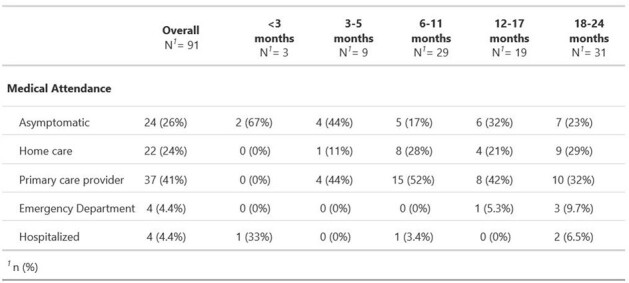

Medical attendance by age group, PREVAIL Cohort, Cincinnati, Ohio, 2017–2020

**Methods:**

Children who were at least 70% adherent to the weekly sample protocol were included in this analysis. Weekly, mid-turbinate nasal swabs were tested using the Luminex Respiratory Pathogen Panel PCR test. Child serum samples were collected at birth, 6 weeks, and 6, 12, 18, and 24 months of age. Symptoms status and medical visits were obtained weekly via SMS text messaging. RSV detection was defined as a swab positive for RSV (A or B) or an increase in RSV pre-fusion log_10_ IgG ( >0.32) or IgA ( >0.20) F-antibody levels. Medical charts were reviewed for unreported medical visits. Asymptomatic infection was defined as no reported fever or cough onset within 7 days of the first PCR positive result.

Figure 1

**Figure:**
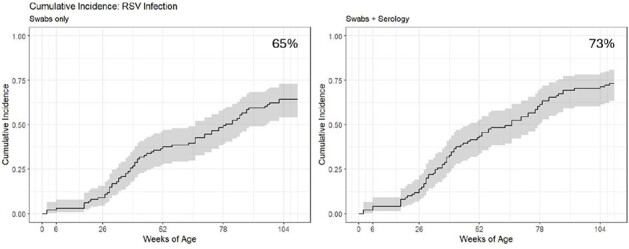

Cumulative incidence of RSV detections by nasal swab and serology, PREVAIL Cohort, Cincinnati, Ohio, 2017–2020

**Results:**

Overall, 137 infections were identified in 74/101 (73%) children: 66% (*n*=91) of infections were identified by PCR (48 (35%) RSV A, 43 (31%) RSV B) and 34% (*n*=46) were identified only by serology (subtype unknown). Two-year cumulative incidence was 65% by PCR only and increased to 73% when including serology; annual incidence was 0.71 infections/child year. Reinfections were common, with 48 (35%) second and 15 (11%) third infections by 2 years of age. Of the 91 PCR-identified infections, 24 (26%) were asymptomatic, 22 (24%) were symptomatic but not medically attended, and 45 (50%) were medically attended, of which 8 (18%) were hospitalized or seen in the emergency department. Seasonality of infection peaked between November and January, although infections were identified in the spring and summer months.

**Figure:**
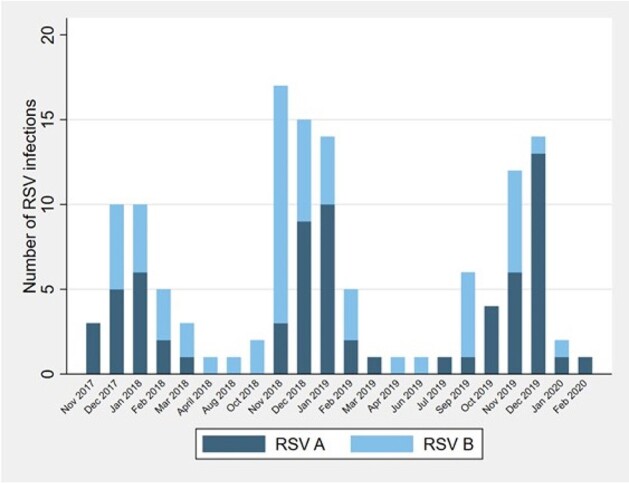

Seasonality of RSV infections throughout the study period (2017-2020) grouped by RSV subtype, PREVAIL Cohort, Cincinnati, Ohio

**Conclusion:**

RSV infections were common in the PREVAIL cohort; most infections were symptomatic, but few were severe. Calculating RSV incidence using only medically attended cases of RSV underestimates disease burden and overestimates the proportion of severe cases.

**Disclosures:**

**Mary A. Staat, MD, MPH**, Cepheid: Grant/Research Support|Merck: Grant/Research Support|Pfizer: Grant/Research Support|Up-To-Date: Honoraria

